# Effect of nitrite treatment on endothelial nitric oxide synthase in human left ventricle biopsy

**DOI:** 10.1186/2050-6511-14-S1-P3

**Published:** 2013-08-29

**Authors:** Jenna Bailey, Sayqa Arif, Jorge Mascaro, Robert S Bonser, Michael P Frenneaux, Melanie Madhani

**Affiliations:** 1 Centre for Cardiovascular Sciences, College of Medical and Dental Sciences, School of Clinical and Experimental Medicine, University of Birmingham, B15 2TT, UK; 2 Queen Elizabeth Hospital Birmingham, University of Birmingham, Birmingham, UK; 3 University of Aberdeen, Aberdeen, UK

## Background

Acute myocardial infarction remains a major cause of morbidity and mortality worldwide. As such, there is a need for improved cardioprotective strategies. Nitrite represents an alternative source of bioactive nitric oxide (NO) particularly during hypoxia [1], and recent studies have demonstrated that nitrite is protective against myocardial ischemia reperfusion (I/R) injury [2]. Several mechanisms of nitrite bioconversion to NO have been proposed, such as xanthine oxidase [3], deoxyhaemoglobin and myoglobin [4], but the cytoprotective effects of nitrite therapy remains to be elucidated. Recent experimental data have shown that mice deficient in endothelial nitric oxide synthase (eNOS) exhibit reduced steady state levels of both plasma and cardiac nitrite levels when compared to the wild-type mice. Thus suggesting that nitrite restores NO bioavailability [5]. Herein, we have investigated whether these effects are translated in man and therefore assessed the effects of nitrite therapy on eNOS phosphorylation and expression in human left ventricle (LV) biopsies.

## Materials and methods

Patients elected for coronary artery bypass grafting (CABG) surgery were subjected to either placebo (saline) or nitrite treatment 10 mcg/kg/min either 30 minutes or 24 hours before surgery. Full thickness LV myocardial free wall tru-cut biopsies were obtained from adjacent non-fibrotic regions just prior to aortic clamping (pre-ischemia) and at 10 minutes post-reperfusion. Biopsies were snap frozen in liquid N_2_ within 1 minute of collection. To determine the effects of nitrite on eNOS expression and phosphorylation status at residue ser1177, Western blot analyses were performed.

## Results

In pre-ischemia LV biopsies, 10 mcg/kg/min nitrite administration 24 hours or 30 minutes before CABG surgery caused a significant increase in eNOS phosphorylation at ser1177 when compared to the placebo group. A similar trend was observed for the expression of total eNOS. LV biopsies taken at reperfusion also demonstrated a significant increase in both total eNOS expression and eNOS phosphorylation at ser1177.

## Conclusion

Nitrite upregulates phosphorylation of eNOS at residue s1177 in patients undergoing CABG surgery. Furthermore, total eNOS expression is also increased in these patients, thus suggesting that nitrite may mediate cardioprotection through eNOS signalling pathway and may potentially restore NO bioavailability.
